# Renal proteomic landscape of a patient with Fanconi renotubular syndrome carrying a novel GATM variant

**DOI:** 10.3389/fphys.2026.1921122

**Published:** 2026-09-23

**Authors:** Fangyi Lu, Luying Yang, Shankui Qian, Chunjie Wang, Feng Feng, Fang Bai, Sha Wang, Li Zong, Ke Zhao, Xiangdong Yang

**Affiliations:** 1 Department of Nephrology, Qilu Hospital, Shandong University, Jinan, Shandong, China; 2 Laboratory of Basic Medical Sciences, Qilu Hospital, Shandong University, Jinan, Shandong, China; 3 Department of Blood Purification, Qilu Hospital, Shandong University, Jinan, Shandong, China

**Keywords:** Fanconi renotubular syndrome, GATM, kidney, proteomics, proximal tubule

## Abstract

**Background:**

Fanconi renotubular syndrome (FRTS) is a rare Mendelian disorder characterized by proximal tubular dysfunction. FRTS type 1 (FRTS1) has been associated with heterozygous variants in *GATM*, encoding mitochondrial glycine amidinotransferase. Although *GATM*-related mitochondrial abnormalities have been proposed to contribute to tubular injury, the molecular alterations in human kidney tissue remain incompletely understood.

**Methods:**

Proteomic profiling was conducted on formalin-fixed paraffin-embedded renal biopsy tissue obtained from a patient with FRTS carrying a novel heterozygous missense variant in *GATM* (NM_001482.3; exon 6: c.949C>T, p.Leu317Phe). Protein abundance was quantified by data-independent acquisition mass spectrometry. Proteins showing marked deviations from control specimens were identified using a Z-score-based approach. Subsequent analyses included Gene Ontology and Kyoto Encyclopedia of Genes and Genomes pathway enrichment, gene set enrichment analysis, gene set variation analysis, and protein-protein interaction network analysis.

**Results:**

Beyond the identification of a novel *GATM* variant, this study provides an exploratory proteomic characterization of renal tissue alterations in a patient with FRTS. Proteomic profiling identified three major groups of altered molecular signatures involving proteasome-related proteins, coat protein complex II-mediated vesicular trafficking-associated proteins, and cytoskeleton- and adhesion-related proteins.

**Conclusions:**

This study represents the first proteomic profiling of FRTS in its native tissue context. The identified proteomic alterations provide an exploratory framework for understanding renal tissue changes associated with FRTS and establish a resource for future validation studies.

## Introduction

Fanconi renotubular syndrome (FRTS) is a proximal tubular disorder characterized by excessive urinary loss of glucose, phosphate, low-molecular-weight proteins, and other solutes normally reabsorbed in this nephron segment (Foreman, 2019). FRTS may occur secondary to systemic diseases or as a primary Mendelian disorder caused by variants in at least five genes: *GATM* (FRTS1), *SLC34A1* (FRTS2), *EHHADH* (FRTS3), *HNF4A* (FRTS4), and *NDUFAF6* (FRTS5) (Magen et al., 2010; Stanescu et al., 2012; Hamilton et al., 2014; Klootwijk et al., 2014; Hartmannová et al., 2016; Reichold et al., 2018; Albuquerque et al., 2023).

Among these subtypes, FRTS1 has been associated with heterozygous variants in *GATM*, which encodes the mitochondrial enzyme glycine amidinotransferase, a key enzyme involved in creatine biosynthesis (Reichold et al., 2018). Patients typically present with generalized proteinuria, glycosuria, phosphaturia, and hypophosphatemia, often accompanied by hypophosphatemic rickets and skeletal deformities. Previous studies have shown that pathogenic *GATM* associated with FRTS1 promote abnormal protein-protein interactions, resulting in linear mitochondrial aggregates within proximal tubular cells. These aggregates impair mitochondrial homeostasis and have been proposed to contribute to inflammation, cellular injury, and renal fibrosis. In contrast, recessive loss-of-function *GATM* variants cause cerebral creatine deficiency syndrome without renal involvement (Reichold et al., 2018; Albuquerque et al., 2023).

Although genetic testing can identify disease-associated variants, it provides limited information regarding the downstream molecular alterations occurring in affected renal tissue. Proteomic profiling offers a complementary approach by characterizing protein-level changes that may reflect the biological consequences of genetic variation and chronic disease processes (Al-Amrani et al., 2021). Compared with *in vitro* or animal models, analysis of human renal tissue may better capture the molecular landscape associated with long-standing tubular injury (Kihlstedt et al., 2025). Such data may facilitate the generation of mechanistic hypotheses and provide a resource for future functional investigations in this rare disease (Meissner et al., 2022).

Here, we describe a family with autosomal dominant FRTS carrying a novel heterozygous missense variant in *GATM*. We further performed proteomic profiling of formalin-fixed paraffin-embedded (FFPE) renal biopsy tissue obtained from the proband to characterize renal tissue alterations associated with this case. To our knowledge, this represents the first proteomic analysis of renal tissue from a patient with FRTS.

## Materials and methods

### Genetic analysis

Genomic DNA was extracted from peripheral blood leukocytes of the proband using a standard DNA extraction kit. Whole exome sequencing was performed on an Illumina HiSeq X Ten platform. Raw sequencing reads were filtered and trimmed using Cutadapt (http://cutadapt.readthedocs.io/en/stable/) to obtain clean reads, which were subsequently aligned to the UCSC hg19 human reference genome using BWA (http://bio-bwa.sourceforge.net/). Variant calling and filtering were performed using the Genome Analysis Toolkit (https://software.broadinstitute.org/gatk/), and variant annotation was conducted using ANNOVAR (http://annovar.openbioinformatics.org/en/latest/). Sequencing quality metrics, including mean sequencing depth and target region coverage, are summarized in **Supplementary Table 1**.

Variants were filtered based on population frequency, sequencing quality, read depth, inheritance information, and predicted functional impact. Variants with a minor allele frequency (MAF) ≥ 0.001 in population databases were excluded, and rare coding variants with adequate sequencing quality and depth were prioritized for further evaluation. Candidate variants were annotated using population databases (gnomAD) and clinical databases (ClinVar), and interpreted according to the American College of Medical Genetics and Genomics (ACMG) guidelines.

The identified heterozygous *GATM* variant was subsequently validated by Sanger sequencing in available family members, including the proband’s father and paternal aunt, to assess segregation (**Figure 1A**).

**Figure 1:**
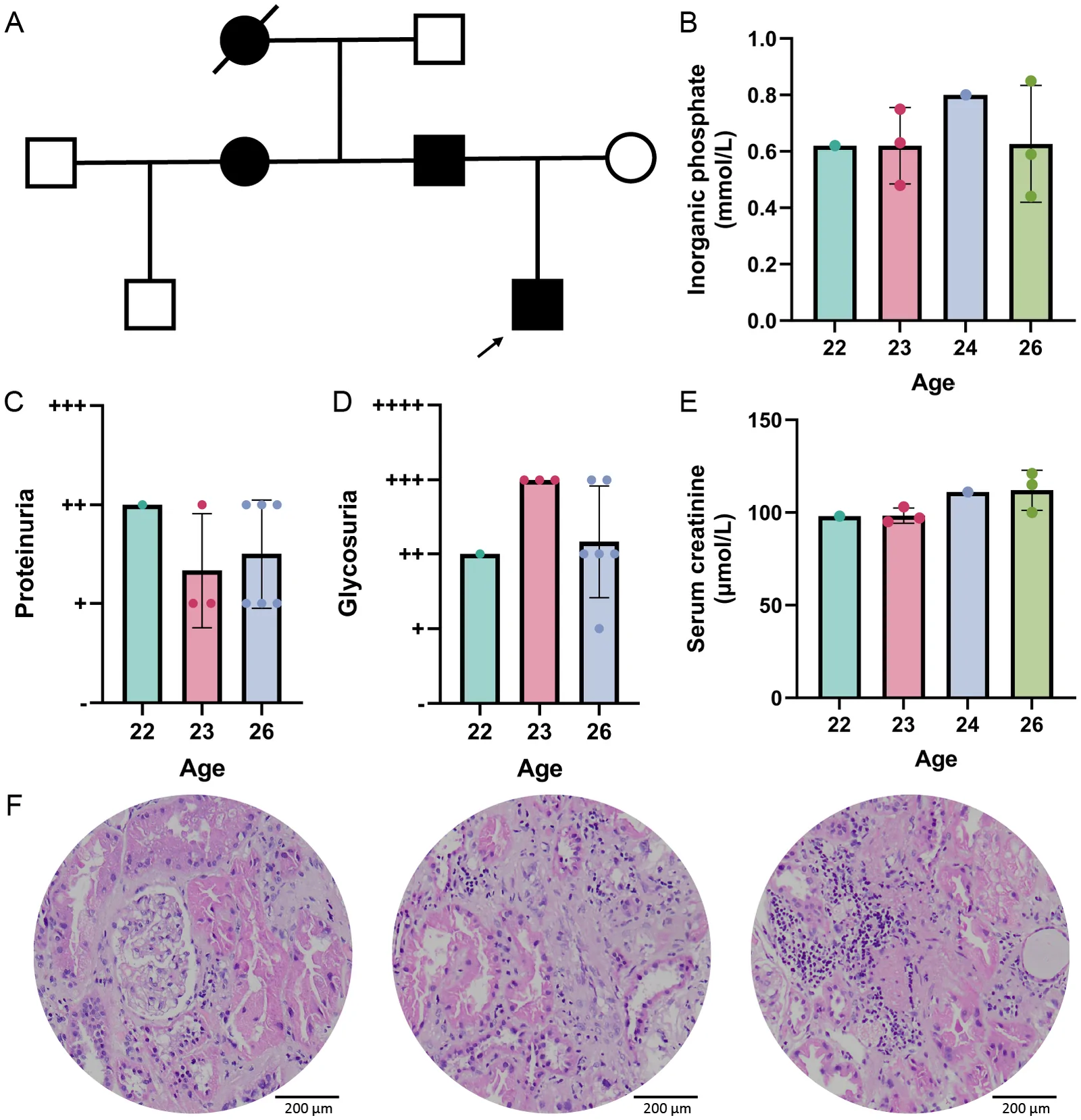
Clinical characterization and renal histopathology of the proband with clinically diagnosed FRTS. **(A)** Family pedigree. The proband is indicated by an arrow. **(B)** Plasma inorganic phosphate concentration of the proband. **(C)** Proteinuria of the proband. **(D)** Glycosuria of the proband. **(E)** Serum creatinine of the proband. **(F)** Representative light microscopy images of the kidney biopsy specimen. Prominent tubulointerstitial injury was observed, including approximately 50% tubular atrophy, epithelial cell swelling, vacuolar degeneration, and focal tubular dilatation with regenerative changes. Interstitial fibrosis involved approximately 50% of the parenchyma, with focal edema and multifocal lymphocytic and monocytic infiltration.

### Study subjects and sample collection

The proband, a 26-year-old male with clinically diagnosed FRTS, underwent percutaneous ultrasound-guided renal biopsy at Qilu Hospital of Shandong University as part of the diagnostic evaluation. Written informed consent was obtained from the patient, and the study was approved by the Ethics Committee of Qilu Hospital, Shandong University (reference number: KYLL-202409-041) and conducted in accordance with the Declaration of Helsinki. A portion of the renal biopsy core was immediately fixed in 10% neutral buffered formalin for 24 h and subsequently embedded in paraffin following standard histopathological protocols. FFPE tissue blocks were stored at room temperature until proteomic analysis.

Control renal tissue samples were obtained from six patients undergoing radical nephrectomy for renal tumors. Histologically normal-appearing renal cortex distant from the tumor lesion was collected from nephrectomy specimens after pathological evaluation. Three patients with minimal change disease (MCD) undergoing diagnostic biopsy (**Supplementary Table 2**). In addition, individual control renal specimens yielded limited amounts of extracted protein, which were insufficient for data-independent acquisition mass spectrometry (DIA-MS) analysis as independent samples. Therefore, nine control samples were combined to generate three pooled control samples, with each pool derived from three independent control specimens. This strategy enabled sufficient protein input for DIA-MS analysis while reducing the influence of inter-individual variability among control tissues. All control samples were processed using the same FFPE protocol as the proband sample.

### Protein extraction and digestion

FFPE tissue sections were deparaffinized with xylene, hydrated with absolute ethanol, and scraped from the slides. An appropriate volume of 25 mM ammonium bicarbonate was added, followed by dithiothreitol (DTT) to a final concentration of 5 mM, and the mixture was heated at 95 °C for 30 min. Sodium dodecyl sulfate (SDS)-free protein lysis buffer was then added, and ultrasonication was applied to facilitate protein solubilization. DTT was supplemented to a final concentration of 10 mM, and the mixture was incubated at 37 °C for 30 min. Iodoacetamide was subsequently added to a final concentration of 55 mM, and the mixture was incubated in the dark for 45 min. The extracted proteins were transferred to a 30 kDa ultrafiltration tube, and the buffer was exchanged to SDS-free lysis buffer by repeated centrifugation at 4,000 × g and 20 °C. The retained protein concentrate was resuspended in 50 mM NH_4_HCO_3_ and digested with trypsin at an enzyme-to-protein ratio of 1:20 (w/w) overnight at 37 °C. After digestion, the peptide solution was collected by centrifugation at 4,000 × g and 20 °C and stored until analysis.

### Liquid chromatography-tandem mass spectrometry analysis

Peptide samples were reconstituted in mobile phase A (H_2_O, 0.1% formic acid) and separated on a Thermo Vanquish Neo UHPLC system equipped with an EASY-Spray™ column (150 μm × 15 cm, Thermo Fisher Scientific, Waltham, MA, USA) using a 12.3 min gradient of mobile phase B (80% acetonitrile, 0.1% formic acid) from 4% to 99% at a flow rate of 1.8-2.5 μL/min. The eluate was directly introduced into an Astral mass spectrometer (Thermo Fisher Scientific, Waltham, MA, USA) with a nanoESI source (ion source voltage, 1.8 kV). Data were acquired in DIA mode. Each sample was analyzed by a single LC-MS/MS injection without technical replication. Full MS spectra were acquired over m/z 380–980 at a resolution of 240,000 (automatic gain control target, 500%; maximum injection time, 5 ms). For DIA fragmentation, the precursor range was divided into 300 consecutive windows, and fragment ions were generated by higher-energy collisional dissociation at a normalized collision energy of 25% (automatic gain control target, 500%; maximum injection time, 3 ms) and detected in the Astral analyzer. Raw DIA data were processed using MSFragger-DIA to generate a spectral library, which was searched against the UniProt *Homo sapiens* reference proteome. Peptide identification and label-free quantification were then performed using DIA-NN with standard parameters (Demichev et al., 2020; Yu et al., 2023). The false discovery rate (FDR) was controlled at 1% at both precursor and protein group levels. Protein intensities were extracted from the DIA-NN output and missing values were assessed in the initial protein abundance matrix. Proteins not detected in the proband sample were excluded from downstream analysis. No missing value imputation was performed because this study focused on patient-specific proteomic alterations rather than conventional group-level differential analysis, and imputation methods may introduce additional assumptions into the estimation of the reference distribution in a limited control set.

### Assessment of control sample heterogeneity

Because control renal tissues were obtained from two different sources, potential source-related proteomic variation was evaluated before downstream comparative analysis. Global proteomic patterns were assessed using principal component analysis (PCA). Pearson correlation analysis was performed to quantify the consistency of different control sources. In addition, protein-level coefficient of variation (CV) was calculated among the three control samples to characterize quantitative variability within the reference control set.

### Reference-based proteomic alteration analysis

Because only one proband sample was available, conventional differential expression analysis methods requiring biological replicates, such as limma or DEqMS, were not applicable. Therefore, a Z-score-based approach was adopted to identify proteins exhibiting extreme deviations from the control reference distribution. Raw protein intensities were log_2_-transformed after adding a pseudocount of 1 to enable transformation of zero-intensity values. For each protein, the mean and standard deviation (SD) of the control samples were calculated based on available quantitative measurements, with missing values in control samples omitted. The Z-score was derived as (patient intensity – control mean)/control SD. Proteins with fewer than two available control measurements among control samples were excluded from Z-score calculation, as these proteins lacked sufficient information for reliable estimation of control variability. This filtering step removed less than 1% of quantified proteins, and the remaining proteins were retained for downstream comparative analysis. Each protein was classified according to both its position relative to the control range and its Z-score. Proteins with patient intensity values exceeding the maximum value observed in all control samples and a Z-score >3 were classified as increased, whereas proteins with patient intensity values below the minimum value of all control samples and a Z-score <−3 were classified as decreased. All other proteins were considered unchanged.

### Functional enrichment analysis

The altered proteins identified above were subjected to functional enrichment analysis using the clusterProfiler package (v4.15.1.2). To account for proteomic detection bias, the background universe was defined as proteins quantified across all samples after quality filtering. Gene Ontology (GO) enrichment was performed separately for biological process (BP), cellular component (CC), and molecular function (MF) ontologies, and Kyoto Encyclopedia of Genes and Genomes (KEGG) pathway enrichment was conducted in parallel. The FDR was controlled using the Benjamini-Hochberg method, with adjusted *P*-value < 0.05 considered significantly enriched. Enrichment results were visualized using ggplot2 (v3.4.2), with selected terms of interest highlighted in bold.

### Visualization of pathway-associated proteins

Proteins associated with KEGG pathways and GO terms that appeared with high frequency among the enrichment results were identified. The overlapping proteins among these categories were extracted, and their log_2_-transformed expression values were visualized as a heatmap using pheatmap (v1.0.12), with row annotations indicating membership in each category and column annotations indicating sample group. Z-scores for these overlapping proteins were additionally displayed as a horizontal bar plot using ggplot2. Protein-protein interaction (PPI) network analysis was performed using the STRING database (https://string-db.org/), and the interaction network was visualized in Cytoscape (v3.10.3).

### Gene set enrichment analysis

To identify pathways coordinately altered at the proteome-wide level, GSEA was performed using the clusterProfiler package. Proteins were ranked according to the log_2_ fold change calculated from the abundance of the proband relative to the mean abundance of the control samples in descending order. The pre-ranked protein list was tested against the C2 curated gene sets (c2.cp.v7.5.1) from the Molecular Signatures Database using the GSEA function with the Benjamini-Hochberg method for FDR control. Enrichment plots for selected gene sets were visualized using the GseaVis package (v0.1.1), with the normalized enrichment score (NES) and *P*-value annotated on each plot.

### Gene set variation analysis

GSVA was performed to assess pathway-level proteomic signatures in the proband relative to control samples. The protein abundance matrix was log_2_-transformed and analyzed against the C2 curated gene sets (c2.cp.v7.5.1) from the Molecular Signatures Database using the GSVA package (v1.46.0). For each pathway, a Z-score was calculated by comparing the proband’s GSVA enrichment score to the mean and SD of the control samples, as described above. Pathways with zero variance among control samples were excluded from Z-score calculation. Pathways were ranked according to their absolute Z-score to identify those showing the greatest deviation from the control reference distribution. Selected pathways of interest were visualized as heatmaps using the pheatmap package, with sample group annotations displayed above the columns.

## Results

### Clinical characterization of the family

A 21-year-old Chinese man presented with bilateral knee pain and was diagnosed with genu valgum. At age 22, he underwent corrective orthopedic surgery. Preoperative laboratory evaluation revealed hypophosphatemia, proteinuria, and normoglycemic glycosuria. At age 26, he was referred to the nephrology service. Repeat biochemical investigations confirmed the above abnormalities and additionally demonstrated low-molecular-weight proteinuria (**Figures 1B–E**). Serological workup for secondary causes was negative, and there was no history of exposure to nephrotoxic drugs or heavy metals. A percutaneous renal biopsy was performed. Light microscopy revealed prominent tubulointerstitial injury, with approximately 50% tubular atrophy, epithelial cell swelling, vacuolar degeneration, and focal tubular dilatation with regenerative changes. Occasional protein casts and calcium salt crystals were present. Interstitial fibrosis involved approximately 50% of the parenchyma, accompanied by focal edema and multifocal lymphocytic and monocytic infiltration. Arteriolar walls were thickened. Glomeruli showed mild ischemic changes, including capillary wall wrinkling and mild segmental thickening, without significant mesangial proliferation or matrix expansion. One of 26 glomeruli exhibited capsular adhesion (**Figure 1F**). Electron microscopy confirmed the overall pathological picture was consistent with diffuse acute and chronic tubulointerstitial injury and glomerular ischemic changes. The diagnosis of FRTS was established based on the constellation of hypophosphatemic rickets, normoglycemic glycosuria, low-molecular-weight proteinuria, tubulointerstitial changes on biopsy, and exclusion of secondary etiologies.

Family history revealed that the proband’s father, a 57-year-old Chinese man, had been diagnosed with unexplained chronic kidney disease (CKD). His serum creatinine was 445 μmol/L, corresponding to an estimated glomerular filtration rate (eGFR) of 12 mL/min/1.73 m², accompanied by proteinuria and non-diabetic glycosuria. Collectively, these findings were consistent with a diagnosis of FRTS. The proband’s paternal aunt, a 43-year-old Chinese woman, also exhibited proteinuria and non-diabetic glycosuria. Her serum creatinine was 87 μmol/L, corresponding to an eGFR of 70 mL/min/1.73 m². The presence of FRTS in three family members across two generations was highly suggestive of an autosomal dominant inheritance pattern.

### Genetic analysis and variant characterization

Whole exome sequencing was performed in the proband to investigate the potential molecular basis of the disease. A novel heterozygous missense variant in *GATM* (NM_001482.3; exon 6: c.949C>T, p.Leu317Phe). The variant was subsequently validated by Sanger sequencing and was found to be present in the proband’s father and paternal aunt. This variant was absent from the gnomAD v4.1.0 population database and was not reported in ClinVar. In silico prediction tools provided supportive evidence for a potentially damaging effect, with SIFT predicting the variant as deleterious (score 0.002) and MutationTaster predicting it as disease-causing (probability score 1.0). According to the ACMG guidelines, the variant was classified as a variant of uncertain significance (VUS), supported by PM2, PP3, and PP4 evidence (**Supplementary Table 3**). No additional candidate variants in other known FRTS-associated genes were identified from the whole exome sequencing dataset (**Supplementary Table 4**).

To date, ten *GATM* variants have been reported in association with FRTS1 (Reichold et al., 2018; Koyun et al., 2023; Kudo et al., 2023; Seaby et al., 2023; Li et al., 2024; Portales-Castillo et al., 2024; Ragate et al., 2024). All previously reported heterozygous variants, with the exception of the recently described p.Gln268Glu (Takayanagi et al., 2026), cluster within the fourth β-sheet (B4) domain (**Figure 2A**). Physiologically, GATM forms homodimers via opposing B2 (**Figures 2B, C**), whereas disease-associated variants create an aberrant B4 interaction interface that drives linear polymerization and insoluble intra-mitochondrial aggregation. Although p.Gln268Glu lies outside B4, it indirectly increases solvent exposure of residues Asp298, Pro299, and Thr339 on the B4 surface, altering its electrostatic properties and shape to similarly induce crystalline aggregation (Takayanagi et al., 2026). This finding reinforces the pathological B4-surface polymerization hypothesis. The p.Leu317Phe variant identified in this study resides within B4 and may promote aggregation through an analogous mechanism (**Figures 2D, E**).

**Figure 2:**
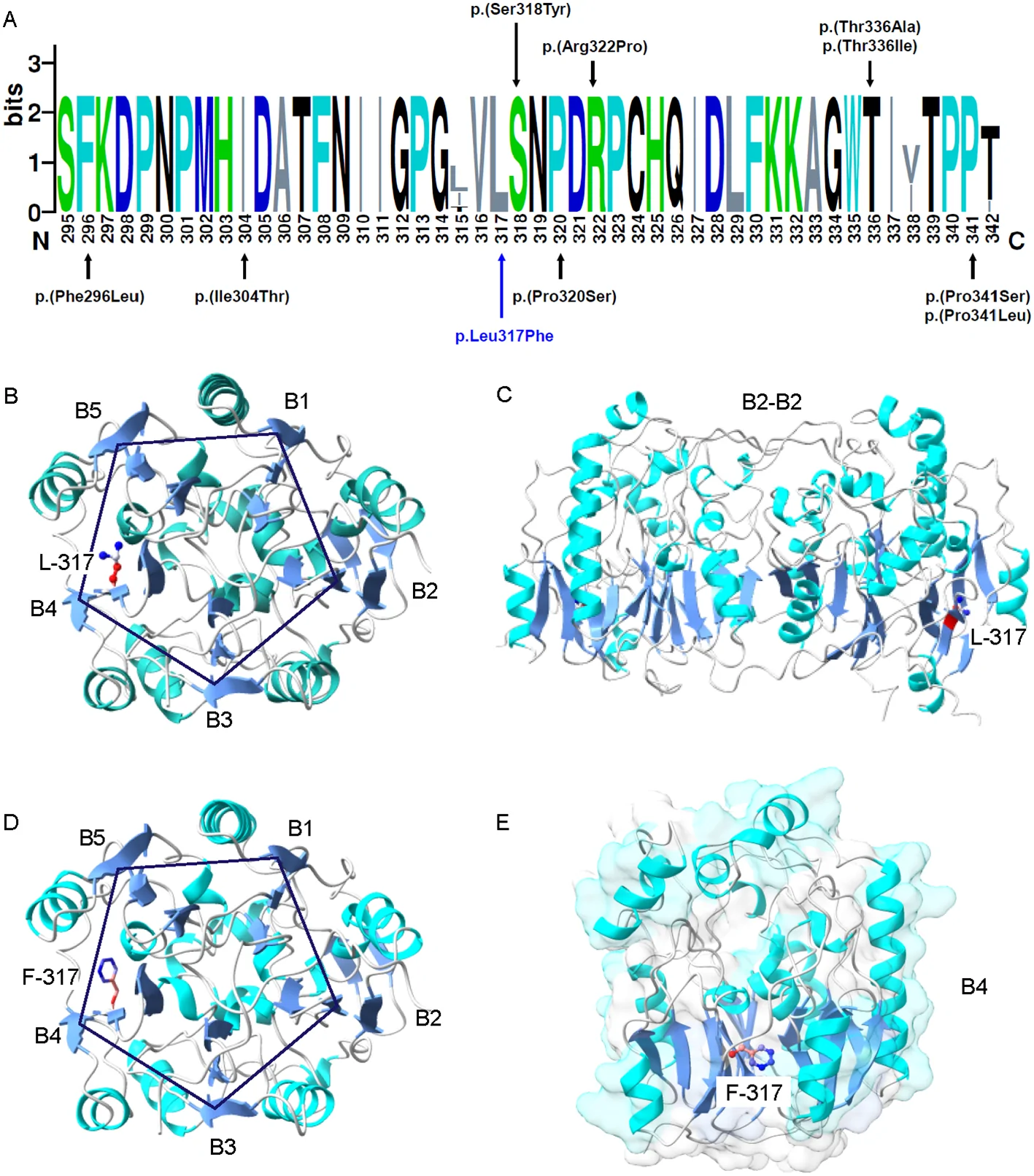
Structural localization of *GATM* variants associated with FRTS1. **(A)** Sequence conservation analysis showing that previously reported *GATM* variants associated with FRTS1 that cluster within the fourth β-sheet (B4) domain. The novel p.Leu317Phe variant identified in this study (highlighted in blue) located within this highly conserved region. **(B)** Schematic representation of wild-type GATM, with five β-sheets (B1-B5) arranged in a fivefold symmetric architecture. **(C)** Wild-type GATM homodimer stabilized by B2-B2 interaction. **(D)** With the exception of the recently reported p.Gln268Glu variant, previously reported GATM variants associated with FRTS1 cluster within the B4 domain. The p.Leu317Phe variant identified in this study is also located within this domain. **(E)** Surface representation of the B4 module of the GATM variant, highlighting the substituted residue Phe317 on the surface of the B4 domain.

### Global proteomic changes in FRTS

DIA-based proteomic profiling was performed on FFPE kidney biopsy tissue. Detailed identification results, including the numbers of identified peptides and quantified proteins across individual samples, are provided in **Supplementary Table 5**. Proteins not detected in the proband sample were excluded. Because control renal tissues were obtained from different sources, the consistency of control proteomic profiles was evaluated before downstream analyses. PCA demonstrated close proximity among the three control samples, whereas the proband sample showed a distinct distribution pattern (**Supplementary Figure 1a**). Pearson correlation analysis and protein-level CV analysis further indicated high consistency and limited quantitative variability among control samples (**Supplementary Figures 1b, c**).

Proteomic alteration analysis using a Z-score-based approach identified altered proteins as those with absolute Z-scores >3 and expression levels outside the range observed in control samples. Overall protein abundance of GATM did not differ between the proband and controls. Given that this patient harbored a heterozygous GATM variant currently classified as a VUS, these findings suggest that the observed molecular alterations may not be primarily attributable to changes in GATM protein abundance, but may instead involve potential effects on protein structure or function. In contrast, multiple fibrosis-associated proteins were markedly increased, particularly extracellular matrix components such as COL1A1 and COL1A2, indicating activation of fibrotic remodeling processes in the affected kidney tissue (**Figure 3A**).

**Figure 3:**
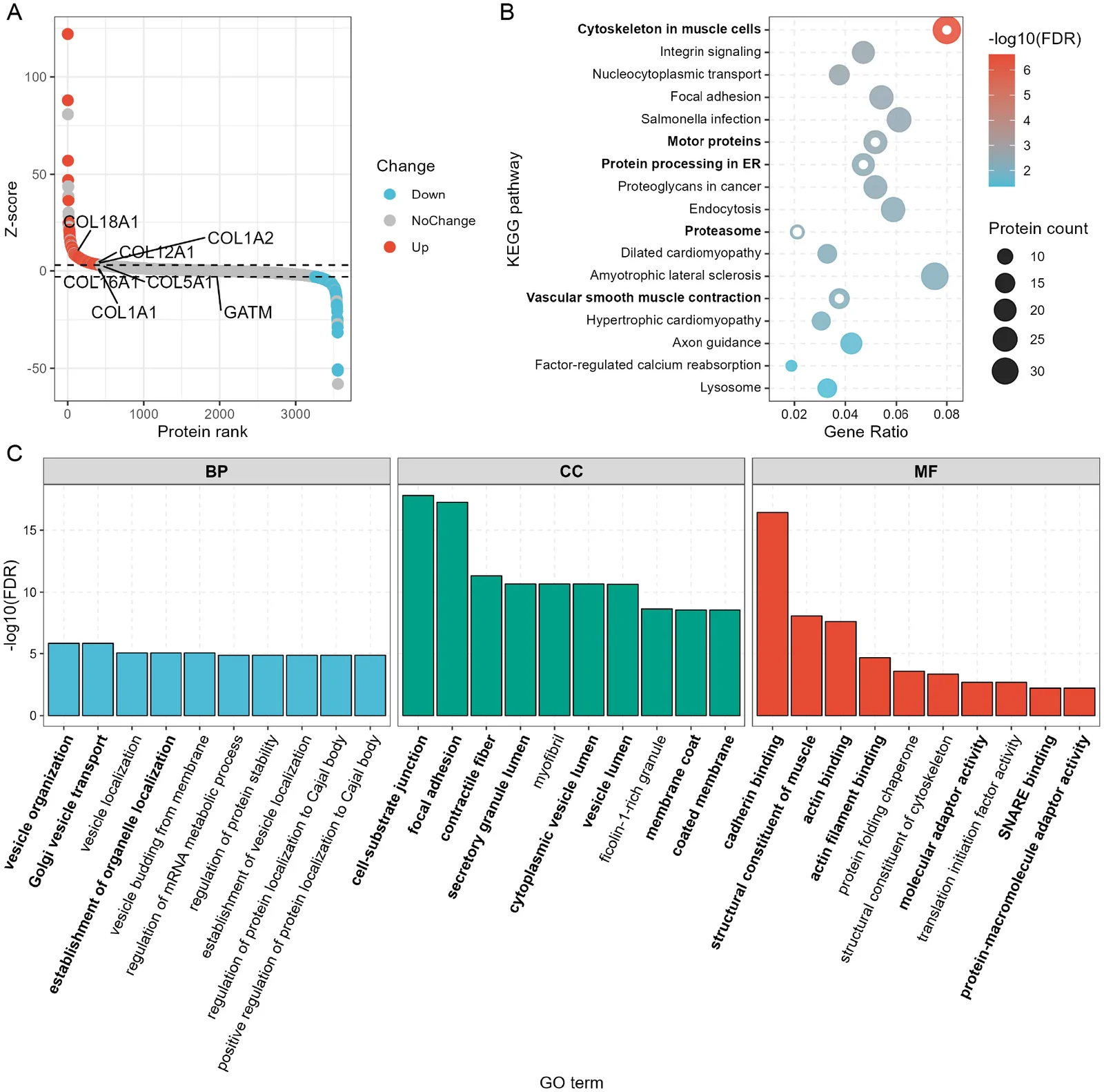
Global proteomic landscape of the proband relative to control samples. **(A)** Distribution of protein-level Z-scores in the proband relative to the control reference. Each point represents one protein, ranked according to its Z-score along the x-axis. Red indicates proteins with increased abundance (patient intensity exceeding the maximum value observed in all controls and Z-score >3), and blue indicates proteins with reduced abundance (patient intensity below the minimum value observed in all controls and Z-score <−3), and gray indicates unchanged proteins. Proteins with |Z-score| >3 that did not satisfy the predefined control-range criterion (i.e., patient intensity did not exceed the maximum or fall below the minimum value observed in all controls) were also classified as unchanged and are shown in gray. Dashed lines indicate the Z = ± 3 thresholds. **(B)** KEGG pathway enrichment analysis of proteins showing marked deviation from the control reference distribution. The x-axis represents the gene ratio (number of query proteins mapped to a pathway divided by the total number of proteins in that pathway), and the y-axis lists enriched pathways. Dot size reflects the number of mapped proteins, and color intensity represents −log_10_(FDR). Hollow circles denote pathways highlighted for downstream analysis, whereas filled circles denote the remaining enriched pathways. Pathways of interest are also shown in bold. **(C)** GO enrichment analysis of proteins showing marked deviation from the control reference distribution, shown separately for BP, CC, and MF. Bar height represents −log_10_(FDR). Terms of interest are highlighted in bold.

Functional enrichment analysis of altered proteins revealed three major biological themes (**Figures 3B, C**). The first theme centered on proteasome, supported by enrichment of the related pathway in KEGG analysis and GO terms. The second corresponded to the endoplasmic reticulum (ER)-Golgi secretory pathway, as reflected by enrichment of protein processing in the ER in KEGG together with vesicle formation and trafficking-related GO terms, such as vesicle organization, Golgi vesicle transport, and SNARE binding. The third theme involved cytoskeleton- and adhesion-associated molecular signatures, highlighted by enrichment of motor proteins-related pathways in KEGG and actin-associated GO terms.

### Proteasome-related alterations in the proband kidney tissue

To further characterize the first functional theme, proteins contributing to the enrichment signals were examined. Among them, PSMD1, PSMB1, PSMA2, PSMD12, and PSMA5 were commonly present across the enriched categories (**Figure 4A**). These proteins are structural and regulatory components of the 26S proteasome complex and play important roles in ubiquitin-dependent protein degradation and maintenance of intracellular proteostasis. Proteomic quantification revealed increased abundance of PSMD1, whereas PSMB1, PSMA2, PSMD12, and PSMA5 were showed reduced abundance in the proband’s kidney tissue compared with controls (**Figure 4B**). PPI analysis demonstrated functional connectivity among these five proteins, supporting their involvement in a coordinated proteasome-associated network (**Figure 4C**). To evaluate global pathway-level alterations, GSEA showed negative enrichment of proteasome-related gene sets (**Figures 4D–F**). Consistently, GSVA demonstrated reduced proteasome-associated pathway signatures in the proband kidney tissue compared with controls (**Figure 4G**).

**Figure 4:**
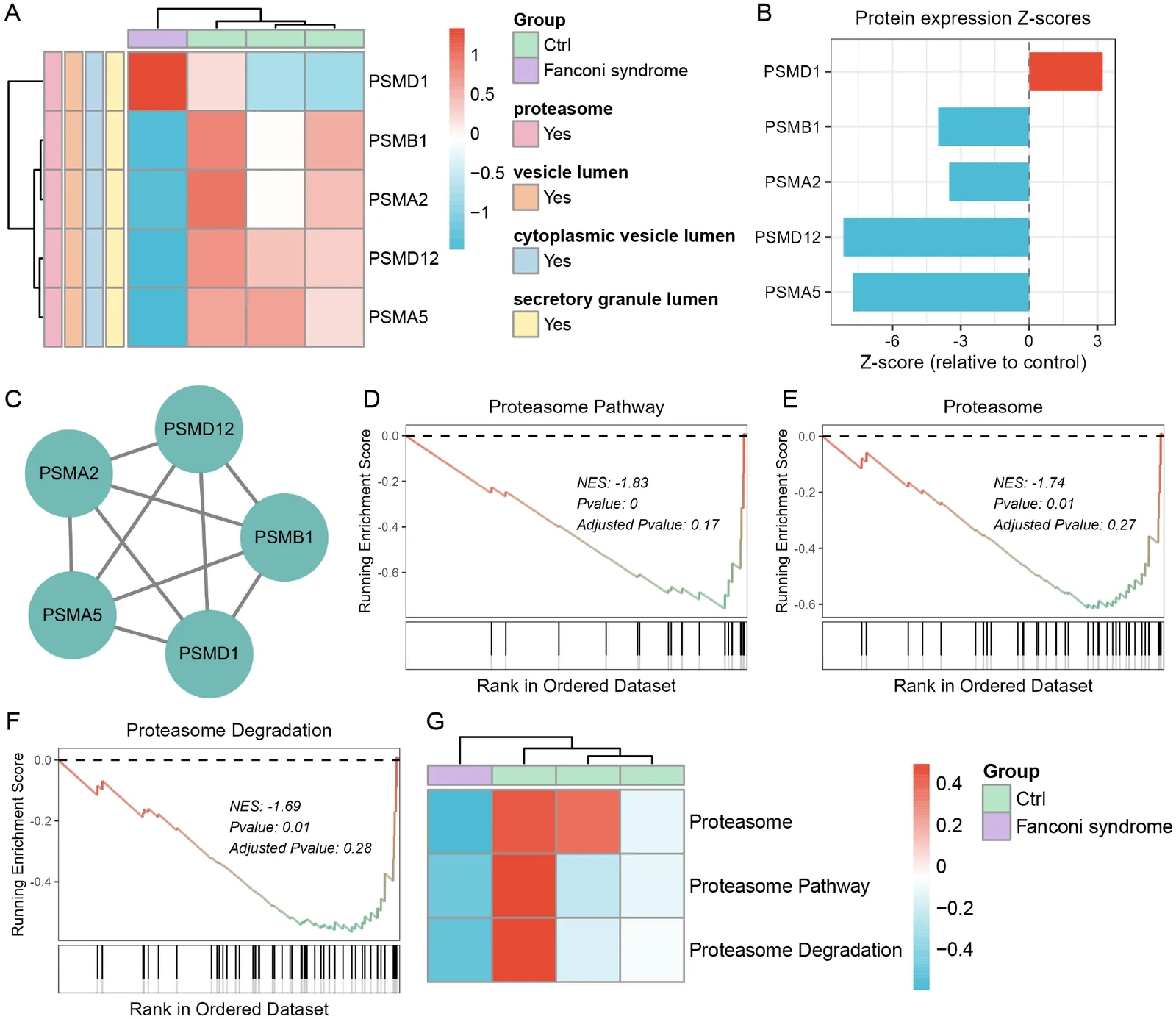
Proteasome-associated alterations identified in the proteomic analysis. **(A)** Heatmap showing the row-wise Z-score-scaled protein abundance of proteins overlapping between the proteasome pathway (KEGG) and vesicle lumen-related GO terms across the proband and control samples. Row annotations indicate pathway membership. Color gradient represents relative protein abundance after row-wise Z-score scaling. **(B)** Horizontal bar plot of protein-level Z-scores for the proteins shown in **(A)**. Red bars indicate proteins with increased abundance, whereas blue bars indicate proteins with reduced abundance. **(C)** PPI network of the overlapping proteins. Node color represents the degree of connectivity, with darker green indicating higher connectivity. **(D–F)** GSEA enrichment plots for selected proteasome-related gene sets: **(D)** Proteasome pathway; **(E)** Proteasome; **(F)** Proteasome degradation. The NES and *P*-value are shown in each plot. **(G)** Heatmap of GSVA pathway scores for proteasome-related pathways across samples. The color gradient ranges from blue (lower pathway score) to red (higher pathway score).

### Coat protein complex II-mediated vesicle trafficking alterations in the proband kidney tissue

The second functional theme, involving ER-Golgi vesicular trafficking, was next investigated. Within the KEGG pathway protein processing in the ER and related GO terms, several overlapping proteins were identified, with those present in at least four categories shown in **Figure 5A**. Proteomic quantification revealed increased abundance of SEC13 and SAR1A, whereas SEC24A, SEC23B, SEC24C, and SAR1B showed reduced abundance in the proband’s kidney tissue compared with controls (**Figure 5B**). These proteins represent key components or regulators of the COPII machinery involved in ER-to-Golgi cargo selection, vesicle budding, and intracellular transport. PPI analysis demonstrated functional connectivity among these proteins (**Figure 5C**). At the pathway level, GSEA showed negative enrichment of the cargo concentration in the ER gene set in the proband kidney tissue (**Figure 5D**). GSVA further revealed reduced pathway scores for both cargo concentration in the ER and COPII-mediated vesicle transport gene sets (**Figure 5E**), consistent with the altered abundance of COPII-related proteins identified above.

**Figure 5:**
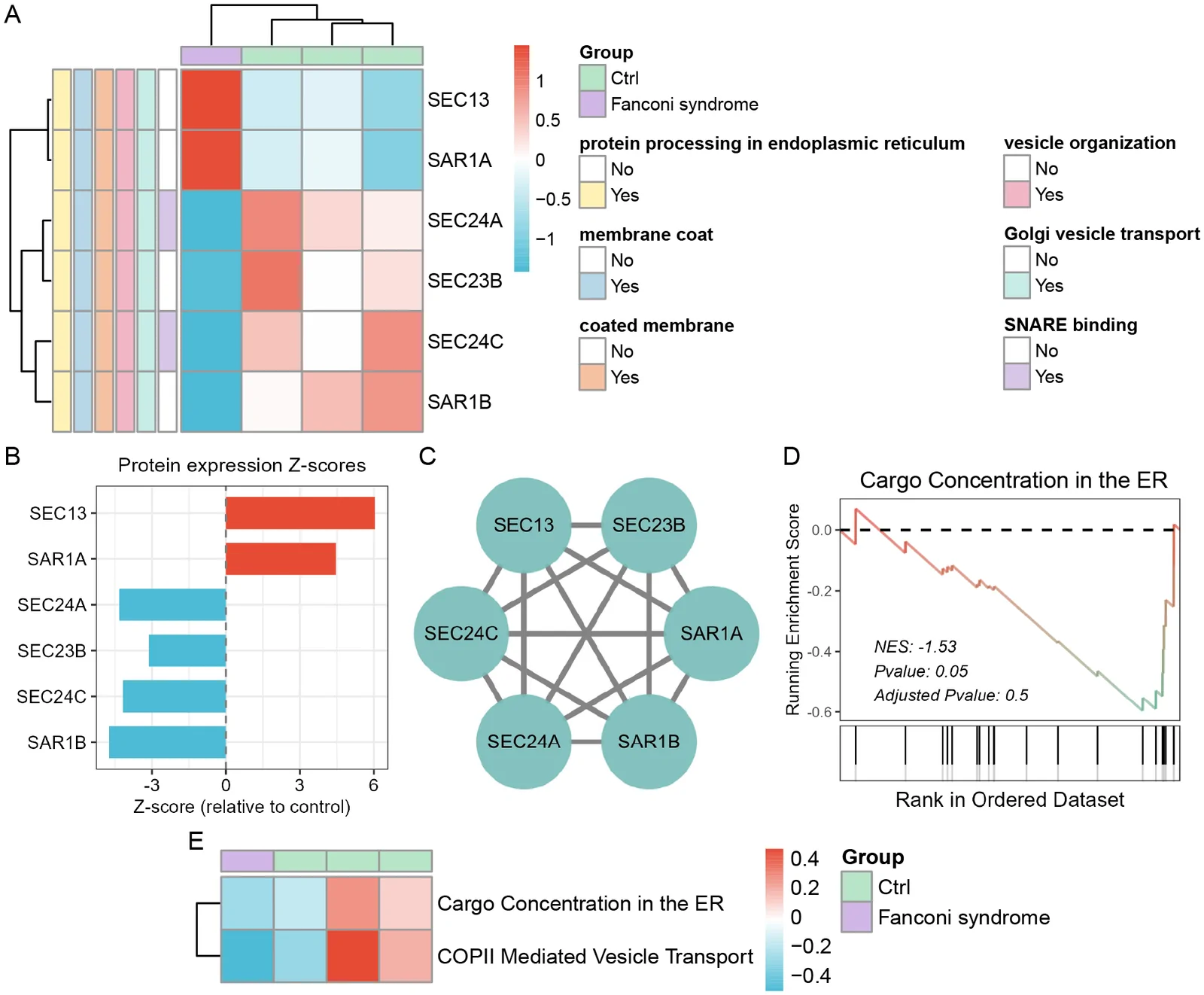
COPII-associated molecular alterations identified in the proteomic analysis. **(A)** Heatmap showing the row-wise Z-score-scaled protein abundance of proteins overlapping among the KEGG pathway protein processing in ER, the GO-CC terms membrane coat and coated membrane, the GO-BP terms vesicle organization and Golgi vesicle transport, and the GO-MF term SNARE binding. Row annotations indicate membership in each category. The color gradient represents relative protein abundance after row-wise Z-score scaling. **(B)** Horizontal bar plot of protein-level Z-scores for the proteins shown in **(A)**. Red bars indicate proteins with increased abundance, whereas blue bars indicate proteins with reduced abundance. **(C)** PPI network of the overlapping proteins. Node color represents the degree of connectivity, with darker green indicating higher connectivity. **(D)** GSEA enrichment plot for the cargo concentration in the ER gene set. The NES and *P*-value are shown in the plot. **(E)** Heatmap of GSVA pathway scores for the cargo concentration in the ER and COPII-mediated vesicle transport gene sets across samples. The color gradient ranges from blue (lower pathway score) to red (higher pathway score).

### Cytoskeleton- and adhesion-related alterations in the proband kidney tissue

The third functional theme related to cytoskeletal organization was further examined. Enriched KEGG pathways (cytoskeleton in muscle cells, motor proteins, and vascular smooth muscle contraction) and multiple cytoskeleton- and adhesion-related GO terms converged on a shared protein cluster. Proteins present in at least six enriched categories are shown in **Figure 6A**. Among them, MYH9 appeared in eleven enriched categories, representing the protein most frequently shared among these functional annotations. Proteomic analysis showed reduced abundance of SYNE1 and increased abundance of VCL, SORBS2, DMD, MYH11, NEXN, TPM2, MYH9, TPM1, and TPM4 in the proband’s kidney tissue (**Figure 6B**). These proteins are involved in actin organization, myosin contractility, and focal adhesion assembly. Notably, several of these proteins, including MYH11 and TPM family members, are also highly expressed in vascular smooth muscle and stromal compartments, suggesting that the observed cytoskeletal-associated signatures may reflect contributions from multiple renal cell populations. PPI analysis demonstrated functional connectivity among them, with VCL exhibiting the highest interaction degree (**Figure 6C**), consistent with its role in linking integrins to the actin cytoskeleton. At the pathway level, GSEA revealed positive enrichment of the muscle contraction gene set (**Figure 6D**). GSVA further indicated increased pathway scores for Rho GTPase activate Rho-associated coiled-coil-containing protein kinases (ROCKs) and protein kinase A (PKA), tight junction, vascular smooth muscle contraction, and muscle contraction gene sets (**Figure 6E**). Together, these findings indicate altered cytoskeleton- and adhesion-associated molecular signatures in the affected kidney tissue. Given the expression patterns of several identified proteins in vascular smooth muscle and stromal compartments, these alterations may reflect changes in tissue composition, vascular remodeling, or epithelial structural responses.

**Figure 6:**
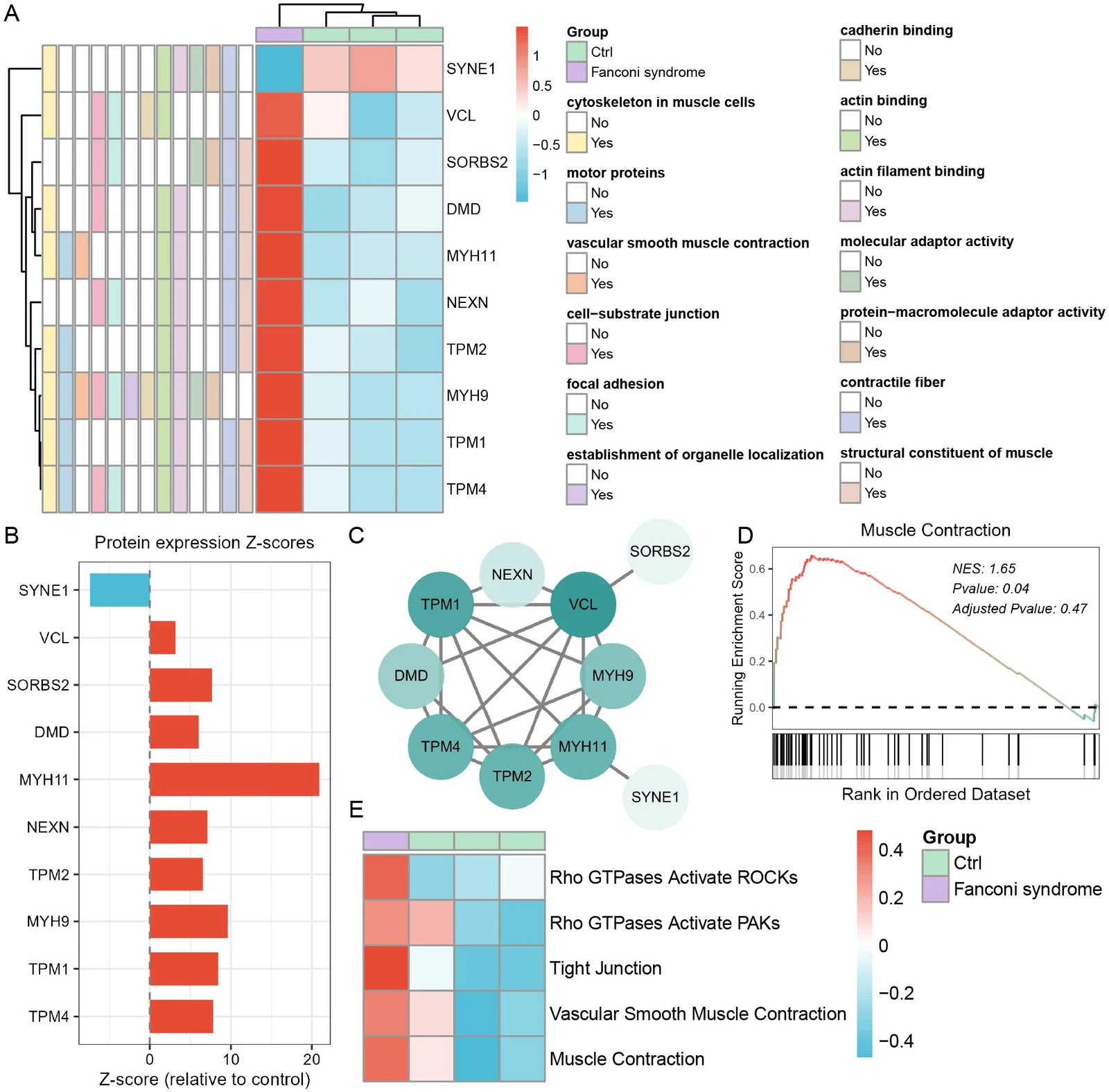
Cytoskeleton- and adhesion-associated molecular alterations identified in the proteomic analysis. **(A)** Heatmap showing the row-wise Z-score-scaled protein abundance of proteins overlapping across the KEGG pathways cytoskeleton in muscle cells, motor proteins, and vascular smooth muscle contraction; he GO-CC terms cell-substrate junction, focal adhesion, and contractile fiber; the GO-BP term establishment of organelle localization; and the GO-MF terms cadherin binding, structural constituent of muscle, actin binding, actin filament binding, molecular adaptor activity, and protein-macromolecule adaptor activity. Row annotations indicate membership in each category. The color gradient represents relative protein abundance after row-wise Z-score scaling. **(B)** Horizontal bar plot of protein-level Z-scores for the proteins shown in **(A)**. Red bars indicate proteins with increased abundance, whereas blue bars indicate proteins with reduced abundance. **(C)** PPI network of the overlapping proteins. Node color represents the degree of connectivity, with darker green indicating higher connectivity. **(D)** GSEA enrichment plot for the muscle contraction gene set. The NES and *P*-value are shown in the plot. **(E)** Heatmap of GSVA pathway scores for cytoskeletal and adhesion-related gene sets across samples. The color gradient ranges from blue (lower pathway score) to red (higher pathway score).

## Discussion

The present study describes a family with autosomal dominant FRTS carrying a novel heterozygous missense variant in *GATM*. Although the identified variant is currently classified as a VUS, this study provides, to our knowledge, the first proteomic characterization of renal biopsy tissue from a patient with FRTS. Proteomic profiling of FFPE kidney tissue revealed three recurrent patterns of protein abundance alterations involving proteasome-associated proteins, COPII-related vesicle trafficking proteins, and cytoskeleton-associated proteins. These exploratory observations extend current knowledge beyond genetic characterization and provide a basis for future studies aimed at validating the molecular mechanisms underlying renal injury in FRTS.

FRTS1 has been proposed to belong to the broader group of proteopathies, in which disease is driven by abnormal protein folding and aggregation (Brockwell and Radford, 2007). Previous studies have shown that pathogenic *GATM* variants can form linear intra-mitochondrial aggregates that disrupt mitochondrial morphology and function (Takayanagi et al., 2026). Maintenance of mitochondrial protein homeostasis depends on multiple quality-control pathways, including the ubiquitin-proteasome system (UPS), which contributes to the degradation of misfolded proteins and the regulation of mitochondrial protein turnover (Margineantu et al., 2007; Radke et al., 2008; Bragoszewski et al., 2013; Bragoszewski et al., 2017). In the present study, proteomic analysis identified altered abundance of several proteasome subunits together with reduced enrichment of proteasome-related pathways in the patient’s renal tissue. Notably, although the proteasome pathway showed an overall negative enrichment pattern, PSMD1, a major non-ATPase subunit of the 19S regulatory particle of the 26S proteasome (Lu et al., 2017), exhibited increased abundance. As a scaffold component involved in proteasome assembly and stability, PSMD1 plays an important role in maintaining proteasome integrity (Schreiner et al., 2008; Budenholzer et al., 2017). The concurrent increase of PSMD1 and reduced proteasome pathway enrichment may indicate a compensatory response to chronic proteotoxic stress, rather than enhanced proteasomal activity. Increased abundance of a single regulatory subunit is insufficient to restore proteolytic capacity, which requires coordinated regulation of multiple proteasome components. Therefore, the observed proteasome alterations may indicate a dysregulated proteostasis network accompanying chronic tubular injury in this case. Whether these changes are directly associated with altered GATM protein or represent secondary consequences of established renal injury remains to be determined by future investigation.

Our proteomic analysis identified alterations in proteins associated with COPII-mediated vesicular trafficking. COPII-mediated transport from the ER to the Golgi apparatus represents a fundamental component of the classical secretory pathway and is essential for the processing and trafficking of a large proportion of cellular proteins (Chatterjee et al., 2021). GSVA indicated suppression of COPII-related pathways in the patient’s renal tissue, suggesting altered secretory pathway homeostasis in renal tubular epithelial cells. Beyond this pathway-level alteration, examination of individual COPII components revealed a non-uniform expression pattern within the trafficking machinery. Specifically, SEC24A and SEC24C were decreased, whereas SEC24B remained unchanged. SEC24 proteins are not merely structural components of COPII coats but also function as cargo-selective adaptors. Previous studies have demonstrated that different SEC24 isoforms possess distinct cargo preferences and selectively regulate the export of specific proteins from the ER (Wendeler et al., 2007). The selective reduction of SEC24A and SEC24C, together with preserved SEC24B expression, may indicate remodeling of COPII cargo-selection pathways.

Importantly, COPII-mediated trafficking and the UPS represent interconnected components of the cellular proteostasis network. While correctly folded proteins are selectively exported from the ER through COPII vesicles, misfolded or unassembled proteins are retained and removed through ER-associated degradation, which ultimately relies on proteasome-mediated degradation (Anelli and Panina-Bordignon, 2019). Thus, the concurrent alterations in COPII components and proteasome-related proteins observed in this patient may represent a broader disturbance of intracellular protein homeostasis. Such dysregulation may be particularly relevant in the context of pathogenic *GATM* variants, which have been associated with mitochondrial protein aggregation and impaired cellular protein homeostasis.

A third finding of our proteomic analysis is the alteration of cytoskeleton-associated proteins and enrichment of pathways related to Rho/ROCK signaling. Renal fibrosis represents a common endpoint of CKD, characterized by excessive extracellular matrix deposition and progressive loss of functional renal parenchyma (Li et al., 2022). Cytoskeletal remodeling and Rho/ROCK signaling have been implicated in the regulation of cellular contractility, mechanotransduction, and fibrogenic responses (Niu et al., 2024; Venugopal et al., 2024). However, several proteins identified in this module, including MYH11 and TPM family members, are also characteristic markers of vascular smooth muscle cells and stromal compartments. Given the limited sampling area of renal biopsy specimens and the presence of arteriolar wall thickening in this patient, differences in vascular content within biopsy cores may contribute to the observed cytoskeletal-associated proteomic changes. Furthermore, because the renal biopsy already exhibited substantial interstitial fibrosis, the current proteomic data cannot determine whether cytoskeletal alterations represent a contributing factor that promotes fibrogenesis or a secondary response to the established fibrotic microenvironment. Therefore, these findings should be interpreted as tissue-level associations between cytoskeletal-related signatures and renal fibrosis rather than evidence of direct causal involvement in tubular epithelial remodeling.

Beyond mechanistic insights, the dysregulated pathways highlighted in this study may provide potential directions for future therapeutic exploration. Among these pathways, Rho/ROCK signaling represents a relatively accessible therapeutic target, as ROCK inhibitors have been investigated in fibrotic diseases (Xie et al., 2020), neurodegenerative disorders (Ye et al., 2024), and ocular diseases (Tan et al., 2025). In addition, therapeutic approaches aimed at restoring protein homeostasis may represent a rational strategy for this type of proteotoxic disorder. Potential approaches under investigation include enhancing molecular chaperone capacity, modulating stress-response pathways, and promoting adaptive protein quality-control mechanisms such as autophagy (Kuzu et al., 2025). In contrast, direct pharmacological targeting of COPII trafficking remains challenging because COPII machinery is essential for global ER-to-Golgi transport and cellular homeostasis.

However, the potential therapeutic implications of these findings should be interpreted cautiously. The molecular alterations identified in this study represent candidate disease-associated pathways rather than validated therapeutic dependencies. In addition, systemic modulation of these pathways may be limited by potential toxicity, given their essential roles in maintaining normal cellular homeostasis. Targeted delivery approaches to proximal tubular epithelial cells may represent a potential strategy to mitigate systemic adverse effects; however, achieving effective and selective delivery of therapeutic agents remains a major challenge in proximal tubular disorders. Systemically administered agents are influenced by multiple biological barriers, including circulation-dependent distribution, glomerular filtration, tubular reabsorption and clearance, and extracellular matrix accumulation during renal fibrosis, all of which may restrict drug accumulation within the target tubular compartment (Zheng et al., 2026). Therefore, even when promising therapeutic targets are identified, achieving sufficient and selective drug exposure in renal tubular epithelial cells while minimizing systemic adverse effects remains a critical obstacle for clinical translation.

We acknowledge several limitations inherent to this study. First, this study represents a single-case deep proteomic analysis of renal biopsy tissue, reflecting the rarity of this clinical condition and the difficulty in obtaining human renal samples. Therefore, the findings should be interpreted as a proof-of-concept exploration of molecular alterations in this individual patient rather than definitive mechanisms applicable to all affected individuals. The use of pooled control samples, although necessary due to limited protein availability from individual control specimens, reduced the ability to assess inter-individual variability and perform conventional statistical comparisons. In addition, the heterogeneous origin of control tissues may have influenced the reference distribution used for Z-score calculation. Given the limited number of pooled controls, missing values and the absence of formal protein-level statistical testing represent inherent limitations of this single-case analysis. Future studies involving larger patient cohorts will be required to validate the identified proteomic signatures using conventional statistical approaches.

Second, the number of quantified proteins was lower than that reported in some kidney proteomic studies, likely due to the limited amount of biopsy-derived FFPE tissue available from the proband, lower protein input compared with pooled controls, and potential effects of FFPE preservation on protein extraction and peptide detection. Nevertheless, this deep proteomic profiling provides a rare opportunity to characterize *in vivo* molecular alterations associated with this disorder. Finally, the heterozygous *GATM* variant identified in this patient remains classified as a VUS. Although the clinical phenotype and proteomic findings suggest potential biological relevance, definitive assessment of pathogenicity will require future functional studies.

In summary, this study provides a deep proteomic characterization of renal tissue from a patient with a rare inherited tubular disorder. Our findings highlight alterations in proteasome-related processes, COPII-mediated vesicular trafficking, and cytoskeleton-associated pathways, suggesting cellular processes that may contribute to tubular injury and fibrosis. These findings expand our understanding of the molecular landscape associated with this disorder and provide potential directions for future mechanistic and therapeutic investigations.

## Data Availability

The mass spectrometry proteomics data have been deposited to the ProteomeXchange Consortium via the PRIDE [1] partner repository with the dataset identifier PXD081180.
