# Supplementary material for: Renal proteomic landscape of a patient with Fanconi renotubular syndrome carrying a novel GATM variant
Source: Front Physiol. 2026 Sep 23;17:1921122. doi: 10.3389/fphys.2026.1921122 (PMC13641987; doi:10.3389/fphys.2026.1921122)
Supplement: Supplementary file 1 [file SupplementaryFile1.docx]

**Supplementary Figures**

**Figure S1.** Assessment of control sample heterogeneity and proteomic consistency.

(a) Principal component analysis (PCA) showing the global proteomic distribution of the Fanconi renal tubular syndrome (FRTS) proband and control samples.

(b) Pearson correlation heatmap showing pairwise correlations among control samples.

(c) Distribution of protein-level coefficients of variation (CV) among the three control samples, reflecting quantitative variability within the control reference set.

**Supplementary tables**

**Table S1.** Quality metrics of whole-exome sequencing data.

| Metric | Value |
| --- | --- |
| Mean sequencing depth | 214.94× |
| Target region coverage ≥10× | 99.73% |
| Target region coverage ≥20× | 99.31% |

**Table S2.** Clinical characteristics of control renal tissue samples used for proteomic analysis.

| Sample ID | Surgical procedure | Diagnosis | Tumor stage (if applicable) | Control tissue location | Age | Sex |
| --- | --- | --- | --- | --- | --- | --- |
| Control 1 | Radical nephrectomy | Clear cell renal cell carcinoma | pT1b | Histologically normal renal cortex distant from tumor | 66 | Female |
| Control 2 | Radical nephrectomy | Clear cell renal cell carcinoma | pT2a | Histologically normal renal cortex distant from tumor | 67 | Male |
| Control 3 | Radical nephrectomy | Clear cell renal cell carcinoma | pT2a | Histologically normal renal cortex distant from tumor | 56 | Female |
| Control 4 | Radical nephrectomy | Clear cell renal cell carcinoma | pT1b | Histologically normal renal cortex distant from tumor | 61 | Male |
| Control 5 | Radical nephrectomy | Clear cell renal cell carcinoma | pT2a | Histologically normal renal cortex distant from tumor | 60 | Male |
| Control 6 | Radical nephrectomy | Clear cell renal cell carcinoma | pT2a | Histologically normal renal cortex distant from tumor | 69 | Male |
| Control 7 | Biopsy | Minimal change disease | NA | NA | 32 | Male |
| Control 8 | Biopsy | Minimal change disease | NA | NA | 54 | Male |
| Control 9 | Biopsy | Minimal change disease | NA | NA | 56 | Male |

**Table S3.** Annotation and interpretation of the *GATM* variant.

| Gene | Transcript | Variant | Protein change | gnomAD frequency | ClinVar annotation | ACMG classification |
| --- | --- | --- | --- | --- | --- | --- |
| *GATM* | NM_001482.3 | c.949C>T | p.Leu317Phe | Not observed | Not  reported | Uncertain |

**Table S4.** Other candidate variants identified after whole-exome sequencing filtering.

| Gene | Transcript | Variant | Protein change | Zygosity | gnomAD frequency | ACMG classification |
| --- | --- | --- | --- | --- | --- | --- |
| *COL4A5* | NM_033380.3 | c.546+92A>G | p.? | Heterozygous | Not observed | Uncertain |
| *PKD1* | NM_001009944.3 | c.7864-8T>C | p.? | Heterozygous | Not observed | Uncertain |
| *JAG1* | NM_000214.3 | c.2518A>C | p.Asn840His | Heterozygous | Not observed | Uncertain |
| *PKP2* | NM_001005242.3 | c.2422del | p.Glu808AsnfsTer79 | Heterozygous | 0.0000544 | Pathogenic |
| *KYNU* | NM_003937.3 | c.766G>T | p.Gly256Ter | Heterozygous | Not observed | Uncertain |
| *PHKB* | NM_000293.3 | c.40_62dup | p.Thr22ArgfsTer106 | Heterozygous | Not observed | Likely pathogenic |
| *IL17RA* | NM_014339.7 | c.1370del | p.Ala457GlyfsTer27 | Heterozygous | Not observed | Likely pathogenic |
| *HOGA1* | NM138413.4 | c.812G>A | p.Arg271His | Heterozygous | 0.0003 | Likely pathogenic |
| *TGM1* | NM000359.3 | c.1193T>C | p.Val398A1a | Heterozygous | Not observed | Likely pathogenic |
| *IFT140* | NM_014714.4 | c.2446C>T | p.Arg816Trp | Heterozygous | 0.0044872 | Uncertain |
| *GJB6* | NM_001110219.3 | c.164C>T | p.Thr55Ile | Heterozygous | Not observed | Uncertain |
| *OPLAH* | NM_017570.5 | c.3523C>T | p.Arg1175Ter | Heterozygous | 0.0000804 | Uncertain |
| *WD R36* | NM_139281.3 | c.2573T>C | p.Leu858Pro | Heterozygous | Not observed | Uncertain |
| *SCN4A* | NM_000334.4 | c.2791_2817del | p.Ser931_Glu939del | Heterozygous | Not observed | Uncertain |
| *UNC13D* | NM_199242.3 | c.2588G>A | p.Gly863Asp | Heterozygous | 0.0044872 | Uncertain |
| *FOXRED1* | NM_017547.4 | c.-31A>G | p.? | Heterozygous | 0.0051348 | Uncertain |
| *TRPM3* | NM_001366145.2 | c.2965A>T | p.Ile989Phe | Heterozygous | Not observed | Uncertain |
| *KANK2* | NM_001136191.3 | c.1848+4C>T | p.? | Heterozygous | 0.0070694 | Uncertain |
| *KANK2* | NM_001136191.3 | c.638A>G | p.GIn213Arg | Heterozygous | 0.000641 | Uncertain |
| *LAMA1* | NM_005559.4 | c.8093C>T | p.Pro2698Leu | Heterozygous | 0.000077 | Uncertain |
| *LAMA1* | NM_005559.4 | c.9172A>G | p.Arg3058Gly | Heterozygous | Not observed | Uncertain |
| *PLXNA2* | NM_025179.4 | c.4208A>T | p.Asp1403Val | Heterozygous | Not observed | Uncertain |
| *TTN* | NM_001267550.2 | c.51268G>A | p.Gly17090Arg | Heterozygous | Not observed | Uncertain |
| *TTN* | NM_001267550.2 | c.63946G>A | p.Val21316Ile | Heterozygous | 0.0000282 | Uncertain |
| *PAD14* | NM_012387.3 | c.1298G>A | p.Ser433Asn | Heterozygous | Not observed | Uncertain |
| *TCF4* | NM_001083962.2 | c.1563G>C | p.Glu521Asp | Heterozygous | Not observed | Uncertain |
| *CAPN3* | NM_000070.3 | c.46G>A | p.Glu16Lys | Heterozygous | 0.0000638 | Uncertain |
| *PRSS56* | NM_001195129.2 | c.1264C>T | p.Arg422Trp | Heterozygous | Not observed | Uncertain |
| *PRSS56* | NM_001195129.2 | c.1522-9C>T | p.? | Heterozygous | Not observed | Uncertain |
| *SETD1B* | NM_001353345.2 | c.1400G>A | p.Arg467G1n | Heterozygous | 0.0000743 | Uncertain |
| *TENM4* | NM_001098816.3 | c. 4895G>A | p.Arg1632His | Heterozygous | 0.0005098 | Uncertain |

**Table S5.** Summary of peptide and protein identification results for individual samples.

| Sample | Identified peptides | Identified proteins |
| --- | --- | --- |
| FRTS | 17566 | 3591 |
| Tumor_normal_1 | 57999 | 6842 |
| Tumor_normal_2 | 36535 | 5452 |
| MCD | 55617 | 6714 |

**Table S6.** Sample-level missing value proportion before filtering.

| Sample | Missing value proportion (%) |
| --- | --- |
| FRTS | 50.25 |
| Tumor_normal_1 | 5.21 |
| Tumor_normal_2 | 24.47 |
| MCD | 6.98 |
